# Whole-Genome Sequences of Staphylococcus aureus Isolates from Positive Blood Cultures

**DOI:** 10.1128/MRA.00898-21

**Published:** 2021-10-28

**Authors:** Itidal Reslane, Margaret Sladek, Paul D. Fey, Baha Abdalhamid

**Affiliations:** a University of Nebraska Medical Center, Department of Pathology and Microbiology, Omaha, Nebraska, USA; University of Rochester School of Medicine and Dentistry

## Abstract

Staphylococcus aureus is a major cause of skin and soft tissue infections as well as bloodstream infections worldwide. Here, we report the draft genome sequences of 18 deidentified S. aureus clinical strains collected from positive blood cultures.

## ANNOUNCEMENT

Staphylococcus aureus is a human skin and mucosa commensal. S. aureus is a leading cause of endocarditis, osteomyelitis, hospital-acquired infections, and bacteremia (1). S. aureus bloodstream infections are a global health concern and are a significant burden on the health care system (2–4). In this study, we announce the whole-genome sequences of 18 clinical S. aureus strains isolated from deidentified positive blood cultures that were collected in December 2020. The S. aureus strains used in this study were deidentified and analyzed anonymously and were therefore exempt from human research committee approval under IRB number 638-21-NH.

These blood samples, previously identified as infected with S. aureus, were streaked onto blood agar plates and incubated overnight at 37°C in a 5% CO_2_ incubator. A single colony was inoculated into 5 ml tryptic soy broth (TSB) and grown aerobically overnight at 37°C with shaking at 250 rpm. Genomic DNA was prepared for sequencing using the Nextera XT DNA library prep kit (Illumina, California) according to the manufacturer’s instructions. The libraries were validated by running 5 μl of PCR cleanup on a 1% agarose gel, bead-normalized using the Nextera XT library normalization beads according to the manufacturer’s instructions, and pooled in equal volumes. The pooled normalized libraries (starting concentration, 2 nM) and PhiX were diluted and denatured according to the MiSeq system user’s guide, with a final concentration of 80 pM. The final pool was heated at 96°C for 3 min to ensure denaturation before sequencing on a MiSeq instrument using a read length of 2 × 300 bp, onboard fastq file generation, and sample demultiplexing, generating 0.6 to 1.4 million paired-end reads per sample. FastQC 0.10, Trimmomatic 0.33, SPAdes 3.12, BBMap 38.06, and QUAST 4.1 were used for examining the quality of sequence reads, trimming the reads, *de novo* assembly, exclusion of contigs smaller than 200 bp, and determining the quality of the *de novo* assembled genomes, respectively (5–9). These bioinformatics tools were used on GitHub (https://github.com/). Default parameters were used for all software unless otherwise specified. The NCBI Prokaryotic Genome Annotation Pipeline (PGAP 5.2) (https://www.ncbi.nlm.nih.gov/genome/annotation_prok/) was used for annotation (10).

The average genome size of the sequenced isolates was 2,801,904 bp, with a range of 2,688,187 to 2,929,278 bp. The average GC content was 32.7%, with a range of 32.81% to 32.63%. The average number of contigs was 47, with a low of 19 contigs and a high of 92 contigs. The highest *N*_50_ value was 973,389 bp, while the lowest *N*_50_ value was 126,572 bp, with the average being 400,216 bp.

### Data availability.

The BioProject accession number for these sequences is PRJNA731492. The GenBank accession numbers, Sequence Read Archive (SRA) accession numbers, numbers of contigs, and *N*_50_ values are provided in Table 1.

**TABLE 1:** Summary characteristics of whole-genome sequencing of Staphylococcus aureus clinical strains isolated from blood cultures^*a*^

Strain	No. of contigs	Largest contig size (bp)	Genome size (bp)	GC content (%)	*N*_50_ (bp)	BioSample accession no.	SRA accession no.^*b*^	GenBank accession no.	No. of reads
IR12	69	548,586	2,735,678	32.81	181,818	SAMN19287364	SRS9065497	JAHKBX000000000	88
IR13	52	1,095,423	2,869,046	32.8	378,757	SAMN19287365	SRS9065498	JAHKBW000000000	184
IR14	40	540,208	2,812,722	32.78	423,370	SAMN19287366	SRS9065499	JAHKBV000000000	171
IR15	48	902,407	2,828,337	32.77	346,657	SAMN19287367	SRS9065500	JAHKBU000000000	171
IR16	36	1,334,846	2,914,446	32.76	513,180	SAMN19287368	SRS9065501	JAHKBT000000000	118
IR21	29	1,035,212	2,829,243	32.76	694,614	SAMN19287369	SRS9065502	JAHKBS000000000	130
IR25	42	690,056	2,834,024	32.74	226,507	SAMN19287370	SRS9065503	JAHKBR000000000	152
IR26	66	304,104	2,742,281	32.73	126,572	SAMN19287371	SRS9065504	JAHKBQ000000000	133
IR31	58	375,313	2,836,112	32.72	225,347	SAMN19287372	SRS9065489	JAHKBP000000000	179
IR32	45	610,153	2,743,060	32.72	207,776	SAMN19287373	SRS9065490	JAHKBO000000000	168
IR33	92	317,269	2,769,668	32.72	149,415	SAMN19287374	SRS9065491	JAHKBN000000000	119
IR35	48	937,961	2,926,696	32.7	380,821	SAMN19287375	SRS9065492	JAHKBM000000000	149
IR36	28	1,043,779	2,741,729	32.69	702,491	SAMN19287376	SRS9065493	JAHKBL000000000	149
IR42	19	1,304,521	2,688,187	32.67	973,389	SAMN19287377	SRS9065494	JAHKBK000000000	156
IR4	68	279,338	2,866,223	32.66	143,163	SAMN19287362	SRS9065487	JAHKBZ000000000	181
IR50	30	1,282,847	2,704,440	32.66	437,176	SAMN19287378	SRS9065495	JAHKBJ000000000	157
IR56	48	895,281	2,929,278	32.65	327,389	SAMN19287379	SRS9065496	JAHKBI000000000	131
IR5	26	1,027,352	2,719,711	32.63	765,444	SAMN19287363	SRS9065488	JAHKBY000000000	172

a Available at NCBI under the BioProject accession number PRJNA731492.

b SRA, Sequence Read Archive.
